# African herbal medicines in the treatment of HIV: *Hypoxis *and *Sutherlandia*. An overview of evidence and pharmacology

**DOI:** 10.1186/1475-2891-4-19

**Published:** 2005-05-31

**Authors:** Edward Mills, Curtis Cooper, Dugald Seely, Izzy Kanfer

**Affiliations:** 1 Department of Clinical Epidemiology & Biostatistics, McMaster University, 1200 Main Street West Hamilton, L8N 3Z5, Canada; 2 Division of Infectious Diseases, University of Ottawa, 501 Smyth Rd., Ottawa, K1H 8L6, Canada; 3 Department of Clinical Epidemiology, Canadian College of Naturopathic Medicine, 1255 Sheppard Ave. East, North York, M2K1M2, Canada; 4 Faculty of Pharmacy, Rhodes University, Grahamstown, South Africa

## Abstract

In Africa, herbal medicines are often used as primary treatment for HIV/AIDS and for HIV-related problems. In general, traditional medicines are not well researched, and are poorly regulated. We review the evidence and safety concerns related to the use of two specific African herbals, which are currently recommended by the Ministry of Health in South Africa and member states for use in HIV: African Potato and Sutherlandia. We review the pharmacology, toxicology and pharmacokinetics of these herbal medicines. Despite the popularity of their use and the support of Ministries of Health and NGOs in some African countries, no clinical trials of efficacy exist, and low-level evidence of harm identifies the potential for drug interactions with antiretroviral drugs. Efforts should be made by mainstream health professionals to provide validated information to traditional healers and patients on the judicious use of herbal remedies. This may reduce harm through failed expectations, pharmacologic adverse events including possible drug/herb interactions and unnecessary added therapeutic costs. Efforts should also be directed at evaluating the possible benefits of natural products in HIV/AIDS treatment.

## 

The use of traditional medicine and Natural Health Products is widespread among those living with HIV infection [1]. Many patients take a broad range of natural health products (NHPs) in addition to their conventional therapeutic products [2-4]. In Africa, traditional herbal medicines are often used as primary treatment for HIV/AIDS and for HIV-related problems including dermatological disorders, nausea, depression, insomnia, and weakness[2,5-8]. Some herbal and traditional medicines are not well-researched, poorly regulated, may contain adulterated products, and may produce adverse effects [8-13]. Notwithstanding these concerns, the use of traditional medicines by Africans living with HIV is believed to be widespread, although insufficiently documented [14-16].

Despite a paucity of evidence on effectiveness, and the possibility of harm, the Ministries of Health of several African nations currently promote traditional medicines for the treatment of HIV and associated symptoms [12,17]. In the case of South Africa, the Ministry of Health is actively promoting the use of traditional medicines with antiretroviral treatments[18].

Two principal African herbal compounds used for HIV/AIDS treatment in sub-Saharan Africa include *Hypoxis hemerocallidea *(common name: African potato), and *Sutherlandia*. These two herbal remedies are currently recommended by the South African Ministry of Health for HIV management [17]. The 14 member states of the South African Development Community (SADC) which includes Angola, Botswana, Democratic Republic of Congo, Lesotho, Malawi, Mauritius, Mozambique, Namibia, Seychelles, South Africa, Swaziland, Tanzania, Zambia, and Zimbabwe, also support their use [19]. Responding to the compelling need for evidence regarding traditional medicines, we reviewed the current evidence for the use of these herbal remedies in HIV care.

## Methods

With the aid of an information specialist, we searched the following databases independently, in duplicate (from inception to December 2004):AltHealthWatch, AMED, CancerLit, CinAhl, Cochrane Controlled Trials Register (CENTRAL), MedLine, and EMBASE. In order to identify unpublished research, we searched *Clinical trials.gov*, National Research Register (UK) and the Meta-Register. Searches were not limited by language. We additionally searched bibliographies of identified reviews and contacted experts in the field. The following search terms were used, but not limited to: "Medicine, African Traditional", "Hypox*", "Sutherlandia", and "HIV."

### Hypoxis hemerocallidea

#### Common names

Magic muthi, yellow stars, star lily, African potato (Eng.); sterretjie, Afrika-patat (Afr.); inkomfe, ilabatheka, sterblom, gifbol, lotsane, molikharatsa[20,21]

*Hypoxis *is a well-known genus of the family Hypoxidaceae. Easily recognizable by its bright yellow star-shaped flowers and strap-like leaves, it has a long history of medicinal use on the African continent. The South African primary health care community is currently using *hypoxis *as an immunostimulant for patients with HIV/AIDS. A daily dose of 2,400 mg of raw plant is purported to be therapeutically effective [22]. Within the genus, two species, *H. hemerocallidea *and *H. colchicifolia *are particularly popular both as African traditional remedies and for the preparation of herbal teas and tinctures.

Rootstocks of this plant have been used by Zulu traditional healers for centuries in the treatment of urinary infections, heart weakness, internal tumors, and nervous disorders [21]. Other unproven uses for this herb include benign prostatic hypertrophy, cancer and hyperglycemia [23-25]. The corms of *H. hemerocallidea *are being used for immune related illnesses such as the common cold, flu, arthritis, cancer and HIV/AIDS. There is some indirect evidence that sterols and sterolins, which are found in the root of *Hypoxis*, have the potential to enhance immunity [26-28]. The popular press in South Africa is promoting preparations of *Hypoxis *as an agent that can boost immunity in HIV/AIDS patients [29,30]. Multiple websites, popular magazines, and even the South African Ministry of Health have supported this assertion [29-32]. Irrespective of the evidence, many Africans claim benefit from eating the root of *H. hemerocallidea *[7,14].

#### Chemical constituents

An important constituent of the plant is a nor-lignan glycoside called hypoxoside, which once in the human gut, readily converts to the aglycone, *rooperol*, a biologically active compound that is purported to have medicinal properties [22,33]. The plant also contains various sterols (β-sitosterol, stigmasterol) and their glycosides (sterolins) such as β-sitosterol glycoside and stanols such as sitostanol also called stigmastanol, which have also been purported to have important biological activity [26,28].

#### Pharmacology and Pharmacokinetics

##### Hypoxoside

Hypoxoside is not absorbed intact into the blood stream. Once in the body, hypoxoside is converted into its aglycone, *rooperol*, a potent antioxidant [34]. This conversion is mediated by beta-glucosidase, an enzyme found predominantly in the gastrointestinal tract. This particular enzyme is released by rapidly dividing cancer cells.

Phase I biotransformation of both *Hypoxoside *and *rooperol *likely occurs via the P450 system and most likely by CYP 3A4 [35]. A multi-dosage trial found only diglucuronide, disulphate, and mixed glucuronide-sulphate metabolites of these two principal constituents in the serum of recipients. Elimination of the metabolites follows first order kinetics with half lives ranging from 20 hours for the two minor metabolites (i.e., diglucuronide and disulphate), to 50 hours for the major metabolite (i.e., the mixed glucuronide-sulphate)[22].

Our group recently reported *Hypoxis*' effect on the P-450 system CYP 3A4 enzyme, the drug transporter P-glycoprotein (P-gp), and the pregnane X receptor (PXR) [35]. *Hypoxis *inhibited up to 86% of the normal CYP 3A4 isoform activity. P-glycoprotein showed moderate activity from exposure to *Hypoxis*, showing 42–51% of the activity strength relative to the known P-gp inhibitor verapamil. Exposure to *Hypoxis *resulted in an almost 2-fold activation of the PXR (p < 0.05). This activation was dose-dependent. Whilst the concentrations used in the *in vitro *experiments were relatively high, the study nevertheless demonstrated that *Hypoxis *possesses the potential to interact with HIV drug metabolizing enzymes, which could subsequently lead to drug resistance, drug toxicity and/or treatment failure. It should be noted, however that this evidence is only from one *in vitro *model and may not translate to the same effect *in vivo*.

#### Toxicity

A Phase I trial in cancer patients failed to establish any clinical, hematological, or biochemical toxicities that could be ascribed to the ingestion of hypoxoside [24]. One recipient did experience an episode of anxiety, nausea, vomiting and diarrhea which was possibly hypoxoside related. The data and safety monitoring committee recently terminated a clinical trial of therapeutic effectiveness in HIV/AIDS patients citing apparent bone marrow suppression [36]. Supporters of this herbal medicine have disputed these inferences [37].

Hypoxoside, when infused in anaesthetized chacma baboons, had no effect on the cardiovascular system, whereas rooperol exerted moderate stimulation during drug administration. The cardiac output increased together with systemic and pulmonary arterial pressures and these changes were not accompanied by changes in heart rate, vascular resistances or in the filling pressures of the heart. These findings are suggestive of increased myocardial contractility, possibly related to rooperol's catechol structure. It is likely that these cardio stimulatory effects will prove to be clinically benign [22]. The molecular basis of rooperol toxicity still needs to be clarified. Biochemical studies have shown that rooperol is a potent inhibitor of leukotriene synthesis in polymorphonuclear leucocytes at a concentration of 1 μM or less [22].

### Sutherlandia Frutescens subspecies Microphylla

#### Common names

Insiswa, Unwele, Mukakana, Phetola, Lerumo-lamadi, cancer bush, kankerbos, kankerbossie [38,39]

#### Background

The flowering shrub *S. frutescens *is a member of the Fabacea family. The recommended therapeutic dose of *Sutherlandia *in humans is 9 mg/kg/day[40]. *Sutherlandia *has been used in the treatment of cancer, tuberculosis, diabetes, chronic fatigue syndrome, influenza, rheumatoid arthritis, osteoarthritis, peptic ulcers, gastritis, reflux esophagitis, menopausal symptoms, anxiety, clinical depression and HIV infection [38,39]. The South African Ministry of Health has concluded that this product is safe based on primate safety studies.

However, scientific data relating to the mechanism whereby Sutherlandia acts on the immune system has not been comprehensively documented. Fernandes *et al *[41] recently described the antioxidant potential of Sutherlandia frutescens where extracts from hot water possessed superoxide as well as hydrogen peroxide scavenging activities which could account for anti-inflammatory properties. In a study by Tai *et al*, [42] ethanolic extracts were shown to have an anti-proliferative effect on several human tumor cell lines but did not show significant antioxidant activity.

Phyto Nova, of South Africa, is the principal distributor of both the powdered and encapsulated forms of this herb, and has attempted to evaluate the purported benefits of this remedy in HIV/AIDS treatment[38]. A definitive conclusion has not yet been reached. Despite the paucity of data, the South African Ministry of Health and member states currently recommend the use of this herbal remedy for HIV/AIDS treatment[17,40].

##### Constituents

The principal constituents of *S. frutescens *purported to be active include L-canavanine, GABA, and D-pinitol. L-canavanine is a non-protein amino acid that is the L-2-amino-4-guanidinooxy structural analogue of L-arginine. There is about 30–40 mg of L-canavanine per dry gram of the *S. frutescens *leaf[38]. D-pinitol is a type of sugar found in many types of legumes and is classified as a chiro-inositol. It is also known as 3-O-methyl-D-chiro-inositol, or 3-0-methyl-1,2,4 cis-3,5,6 *trans*-hexahydroxy-cyclohexanol. GABA (gabba-amino butyric acid) is both an amino acid and inhibitory neurotransmitter. It is found at levels of 14 mg per gram dry leaf of *S. frutescens*[38].

One of the chemical constituents of Sutherlandia, L-canavanine, is an arginine analogue. L-canavanine has been reported to have anti-viral activity against influenza and retroviruses, including HIV [43]. A US patent registered in 1988 claimed that 95% of HIV-infected lymphocytes were selectively destroyed *in vitro*. Unfortunately, no further studies of the effect of this herb on HIV have confirmed this claim. D-pinitol another important constituent of Sutherlandia has also been suggested for the treatment of wasting in cancer and AIDS patients although evidence is scant[44].

#### Pharmacokinetics and pharmacology

The pharmacokinetic properties of Sutherlandia have largely not been assessed[40]. We have demonstrated *in vitro *effects of *Sutherlandia *on CYP3A4, P-gp, and PXR [35]. *Sutherlandia *produced near complete inhibition of CYP3A4 (96%). P-gp activity was moderate under exposure of *Sutherlandia*, showing 19–31% of the activity strength relative to verapamil. A PXR assay demonstrated a greater than 2-fold activation with exposure to *Sutherlandia *which was dose-dependent (P < 0.01). Once again, in spite of the relatively high concentrations used in the *in *vitro experiments, these results tentatively suggest that human consumption of *Sutherlandia *could affect antiretroviral drug metabolism leading to bi-directional drug interactions and loss of therapeutic efficacy. *In vivo *human studies are required to determine if there is a clinically relevant drug/herb interaction and if so what the true extent of the interaction is.

#### Toxicity

*Sutherlandia *has a relatively long history of seemingly safe usage in Africa. Known side effects include occasional mild diarrhea, dry mouth, mild diuresis, and dizzyness in cachectic patients [38,39]. An extensive toxicology screening in a primate model using dosages up to 9 times greater than the recommended dose of 9 mg/kg/day did not identify clinical, hematological or physiologic toxicity with *Sutherlandia *[45].

L-canavanine may be associated with important toxicities including a systemic lupus erythematous syndrome [46]. The non-protein amino acid can be incorporated into protein in place of arginine and may, after long term usage, result in autoimmunity [47,48]. Rare reports of teratogenicity and induction of abortion exist [49].

## Discussion

The widespread use of herbal compounds by Africans living with HIV/AIDS should be of concern to clinicians and policy makers. Clearly, patients will continue to access traditional healing systems as it is important to local cultural values and beliefs. Therefore, efforts should be made by mainstream health professionals to provide validated information to traditional healers and patients on the judicious use of herbal remedies. This may reduce harm through failed expectations, pharmacologic adverse events and unnecessary added therapeutic costs. Efforts should also be directed at evaluating the possible benefits of natural products in HIV treatment.

It is not unreasonable to suggest that some products may have therapeutic benefits as examples from history and the recent past have provided us with effective anti-malarials [50] and cancer treatments[51]. Indeed, some of the earliest forms of protease inhibitors were derived from natural products [52-54]. Efforts should be directed at determining the therapeutic efficacy of these remedies as well as the possibility of interactions through systematic research and clinical trials.

Several studies have highlighted key problems related to primary care delivery by traditional healers in Africa [8,55,56]. Key issues include hygiene, toxicity and financial cost. Traditional healers have been implicated in the spread of blood borne diseases including HIV and other infectious disease by the re-use of medical instruments and lack of hand washing [8,55,56]. Prescriptions to take toxic plants for HIV treatment have also resulted in severe adverse events, including death[56]. Recent policy efforts have recognized the substantial use of traditional medicines and several African nations have included traditional healers in educational campaigns in order to instruct them on safe and hygienic practices, condom distribution and knowledge dissemination[2,5,6,14,57].

Despite the relatively high concentrations of herbals used in our *in vitro *work, the results serve as a warning and suggest that biologically active constituents of these herbal remedies clearly may have an effect on HIV drug metabolism as a result of their inhibitory activity on enzymes and efflux drug transporter systems. These results highlight the need for *in *vivo investigations and circumspection when utilizing herbal drugs as routine care for HIV patients and underscores the need for clinical studies in humans to unveil any possible drug interaction of these herbal agents with antiretrovirals. Failure to do this may result in bi-directional drug interactions that may put patients at risk for treatment failure, viral resistance or drug toxicity.

Cultural values are an inherent part of healthcare and an important component of practicing evidence-based healthcare [58]. In the context of HIV treatment in Africa, patients often choose traditional healing systems as primary care. This, coupled with the difficulties in accessing antiretroviral treatment, justify further efforts to determine the scope of traditional medicine use, identify the negative consequences of this practice and evaluate the benefits of herbal remedies. In addition, it is important to understand the values of those providing mainstream healthcare and those practicing traditional medicine as their perspectives provide highly relevant social inferences and should be interpreted with an attempt to understand their cultural worldviews and practices.

In conclusion, given the Global Fund's recent announcement of funds to make anti-retroviral therapy widely available in Africa, and the South African Ministry of Health, along with member states and NGO's endorsement of the use of traditional African herbs such as *Hypoxis *and *Sutherlandia *as HIV/AIDS remedies [17], initiating policy on herbal medicines should be based on research evidence. Efforts are required to determine the safety, efficacy and pharmacological profile of the many herbal compounds used in Africa. Collaboration with traditional healers is justified to fully understand what remedies are in use for HIV and to educate those providing alternative medical services against unsafe practices.
